# Cancer-control outcomes of patients with metastatic hormone-sensitive prostate cancer and ≥ 10 bone metastases receiving apalutamide: a real-world cohort

**DOI:** 10.1186/s12885-026-15803-y

**Published:** 2026-03-02

**Authors:** Mike Wenzel, Maximilian Filzmayer, Carolin Siech, Quynh Chi Le, Benedikt Lauer, Lena Theissen, Benedikt Hoeh, Hans Heinzer, Markus Graefen, Thomas Steuber, Maximilian Kriegmair, Felix K.-H. Chun, Philipp Mandel

**Affiliations:** 1 Department of Urology, Goethe University Frankfurt, University Hospital, Frankfurt, Germany; 2 Martini-Klinik Prostate Cancer Center, University Hospital Hamburg-Eppendorf, Hamburg, Germany; 3 Urological Clinic Munich-Planegg, Planegg, Germany; 4 Department of Urology and Urosurgery, Medical Faculty Mannheim, University Medical Center Mannheim, Heidelberg University, Mannheim, Germany

**Keywords:** Metastatic prostate cancer, Survival, mHSPC, Apalutamide, Bone metastases

## Abstract

**Purpose:**

Currently available post-hoc TITAN study data indicate favorable cancer-control in metastatic hormone-sensitive prostate cancer (mHSPC) patients treated with apalutamide, even in patients with high metastatic burden, such as ≥ 10 bone metastases. However, these findings have never been validated in real-world setting.

**Patients and methods:**

We relied on the FRAMCAP (FRAnkfurt Metastatic Cancer database of the Prostate) and stratified apalutamide-treated mHSPC patients according to number of bone metastases (≥ 10 vs. < 10). Primary endpoints were time to metastatic castration-resistant prostate cancer (ttCRPC) and overall survival (OS). Finally, exploratory analyses were made against mHSPC treatment with abiraterone and docetaxel.

**Results:**

Of 105 apalutamide-treated mHSPC patients, median age was 71 years and median PSA 46 ng/ml. In total, 23% of included patients had ≥ 10 bone metastases. Patients with ≥ 10 bone metastases harbored higher PSA level at treatment start (254 vs. 29 ng/ml) and achieved less PSA response under treatment (PSA nadir 0.64 vs. 0.03 ng/ml, both *p* < 0.01). Regarding ttCRPC, no statistically significant difference was observed between ≥ 10 vs. < 10 bone metastases with median ttCRPC of 32 vs. 37 months (*p* = 0.15). Regarding OS, median OS was significantly shorter for ≥ 10 vs. < 10 bone metastases (29 vs. 64 months, hazard ratio: 2.5, *p* = 0.02), even after multivariable adjustment for baseline patient and tumor characteristics. In further analyses, apalutamide (32 months) showed numerically longer median ttCRPC compared to abiraterone (18 months) and docetaxel (16 months) in patients with ≥ 10 bone metastases.

**Conclusion:**

In real-world setting, apalutamide-treated mHSPC patients presenting with a high bone metastatic burden achieve virtually similar ttCRPC outcomes compared to those with a lower metastatic burden. Exploratory comparisons with other first-line doublet mHSPC treatment options indicate a potential advantage of apalutamide.

**Clinical trial registration:**

Not applicable.

**Supplementary Information:**

The online version contains supplementary material available at 10.1186/s12885-026-15803-y.

## Introduction

 Metastatic hormone-sensitive prostate cancer (mHSPC) represents a heterogeneous disease with varying prognoses depending on disease burden and metastatic sites [1]. Bone metastases are the most common type of metastases and a critical determinant of survival and treatment response, with a higher metastatic burden generally associated with worse outcomes [1–5]. Androgen receptor pathway inhibitors (ARPI) have become the standard treatment for mHSPC within the recent years [6]. Currently available ARPI doublet treatment options are enzalutamide, apalutamide, abiraterone or darolutamide [7–15].

The TITAN trial demonstrated that the doublet therapy of apalutamide with ADT significantly improved cancer-control rates in mHSPC, achieving over 40% of patients with ultra-low PSA nadir ≤ 0.02 ng/ml [2, 10, 16, 17]. However, patients with an extensive bone metastatic burden pose a unique clinical challenge due to their aggressive disease biology and poorer prognosis [1–5]. Therefore, triplet therapy consisting of ARPI plus taxane-based chemotherapy and ADT with potentially increased risk of adverse events and toxicity may be considered for patients with high-volume disease according to CHAARTED criteria [6, 11–13, 18–21]. A recently published post-hoc analysis of the TITAN trial specifically evaluated the efficacy of the doublet therapy of apalutamide with ADT in patients with more than 10 bone metastases [22]. The analysis revealed that the doublet therapy reduced the risk of death by 46%, compared to ADT alone.

Given these findings, intensification of systemic therapy with apalutamide to ADT as a doublet therapy appears to offer meaningful clinical benefits for patients with mHSPC even with high bone metastatic burden without increasing the risk of the toxicity due to taxane-based chemotherapy. However, real-world data on cancer-control outcomes in mHSPC patients stratified by bone metastatic burden are lacking.

We addressed this knowledge gap and relied on the FRAnkfurt Metastatic CAncer database of the Prostate (FRAMCAP) to investigate the association between the number of bone metastases and cancer-control outcomes in apalutamide-treated mHSPC patients, focusing on time to metastatic castration-resistant prostate cancer (ttCRPC) and overall survival (OS). In addition, we exploratorily compared outcomes in patients with extensive bone metastatic burden treated with apalutamide to those receiving other approved doublet therapies.

## Materials and methods

### Study population

After obtaining approval from the local ethics committee and adhering to the principles of the Declaration of Helsinki, we retrospectively identified all mHSPC patients treated with apalutamide from the FRAMCAP database. The FRAMCAP database prospectively samples all metastatic prostate cancer patients treated at the Department of Urology of the University Hospital Frankfurt (Germany) and discussed within a multidisciplinary tumor board since 2014. The inclusion criteria for this study required patients to have mHSPC status with at least one bone metastasis and availability of the number of bone metastases on staging prior to treatment initiation. Staging was performed either conventionally with CT scan plus bone scan or with PSMA-PET-CT. As a result of these criteria, 105 mHSPC patients treated with apalutamide qualified for study inclusion. According to the post-hoc analysis of the TITAN phase III study cohort with favorable cancer-control rates also for patients with more than 10 bone metastases, the study cohort was divided into two groups: ≥ 10 vs. < 10 bone metastases [22]. Concomitant metastases such as lymph nodes or visceral metastases were allowed. For exploratory analyses with other mHSPC treatments, we additionally included 17 abiraterone-treated and 24 docetaxel-treated mHSPC patients with ≥ 10 bone metastases.

### Study endpoints

The main study endpoints were ttCRPC and OS. TtCRPC was defined as time from initiation of therapy for mHSPC to the development of metastatic castration-resistant prostate cancer (mCRPC), subsequent systemic treatment date or death. OS referred to the time from treatment initiation to death. mCRPC was defined according to EAU guidelines [6, 23]: Three consecutive rises of PSA values during mHSPC treatment with a PSA level above 2 ng/ml and 50% rise above the nadir. Moreover, radiographic progression of two new osseous or one soft tissue metastasis using RECIST (Response Evaluation Criteria in Solid Tumors) were considered as new mCRPC status [24].

### Statistical analyses

Descriptive statistics encompassed frequencies and proportions for categorical variables. Medians and interquartile ranges (IQR) were reported for all continuously coded variables.

To assess group differences, the Wilcoxon rank sum test was used for continuous or ordinal variables without normal distribution. Pearson’s Chi-squared test was applied to evaluate differences in proportions for categorical variables. Fisher’s exact test was used in cases with small frequencies. For all analyses, Kaplan-Meier curve-derived estimates with log-rank tests, as well as univariable and multivariable Cox regression models, were applied. For ttCRPC analyses, multivariable Cox regression model adjustment was made for age at mHSPC diagnosis, Eastern Cooperative Oncology Group (ECOG) performance status, de novo vs. recurrent mHSPC and Gleason score. For OS analyses in multivariable Cox regression models, additional adjustment for number of received therapy lines for metastatic prostate cancer was performed. Variables were selected a priori based on established clinical relevance and low collinearity, while additional correlated variables were excluded to avoid model overfitting [25].

All tests were two sided with a level of significance set at *p* < 0.05. R software environment for statistical computing and graphics (version 3.4.3) was used for all analyses. Analyses were conducted using available data only. No imputation of missing values was performed, and all statistical analyses were based on complete-case methodology for the respective variables. Therefore, denominators may vary across analyses due to missing progression or follow-up data.

## Results

### Study population and baseline characteristics

Overall, 105 bone metastatic mHSPC patients treated with apalutamide could be included for the current analyses (Table 1). Median age at mHSPC stage was 71 years (IQR: 64–77) with a median PSA level of 46 ng/ml (IQR: 14–120 ng/ml). In the overall cohort, the PSA nadir under apalutamide treatment was 0.06 ng/ml (IQR: 0.01–0.58 ng/ml).

**Table 1:** Descriptive characteristics of 105 metastatic hormone-sensitive prostate cancer patients treated with apalutamide, stratified according to ≥ 10 vs. < 10 bone metastases

Characteristic	*N*	Overall *N* = 105^a^	≥ 10 bone metastases *N* = 24 (23%)^a^	< 10 bone metastases *N* = 81 (77%)^a^	*p*-value^b^
Age at metastatic PCa (years)	104	71 (64, 77)	72 (67, 79)	70 (64, 77)	0.4
PSA at mHSPC (ng/ml)	95	46 (14, 120)	254 (84, 531)	29 (11, 91)	< 0.001
PSA nadir at mHSPC (ng/ml)	71	0.06 (0.01, 0.58)	0.64 (0.08, 3.91)	0.03 (0.01, 0.17)	0.001
PSA response ≥ 90%	68	67 (99%)	16 (100%)	51 (98%)	> 0.9
PSA at mCRPC (ng/ml)	26	8 (2, 20)	16 (7, 20)	4 (1, 20)	0.2
Systemic therapy lines for PCa	105	2.00 (2.00, 2.00)	2.00 (2.00, 2.00)	2.00 (2.00, 2.00)	0.7
ECOG status	87				0.5
0		62 (71%)	14 (67%)	48 (73%)	
1		18 (21%)	4 (19%)	14 (21%)	
≥ 2		7 (8.0%)	3 (14%)	4 (6.1%)	
Cardiovascular disease	102	35 (34%)	9 (38%)	26 (33%)	0.7
Gleason score 8–10	100	72 (72%)	17 (77%)	55 (71%)	0.5
De Novo mHSPC	104	96 (92%)	24 (100%)	72 (90%)	0.2
Local therapy with RP/RT	105	31 (30%)	1 (4.2%)	30 (37%)	< 0.01
MDT	99	17 (17%)	0 (0%)	17 (22%)	0.01
Concomitant visceral metastasis at mHSPC	101	5 (5.0%)	1 (4.5%)	4 (5.1%)	> 0.9
High-volume mHSPC	94	41 (44%)	21 (100%)	20 (27%)	< 0.001
High-risk mHSPC	97	56 (58%)	22 (100%)	34 (45%)	< 0.001
Treatment of mCRPC	105				0.7
Chemotherapy		14 (13%)	2 (8.3%)	12 (15%)	
ARPI		15 (14%)	5 (21%)	10 (12%)	
Lu-PSMA		1 (1.0%)	0 (0%)	1 (1.2%)	
Radium		1 (1.0%)	0 (0%)	1 (1.2%)	
None/Other/NA		74 (70%)	17 (71%)	57 (70%)	

^a^ Data are presented as median (IQR) or n (%)

^b^ Wilcoxon rank sum test, Pearson’s Chi-squared test, Fisher’s exact test

*Abbreviations*: *ARPI *Androgen receptor pathway inhibitors, *ECOG *Eastern Cooperative Oncology Group, *IQR *Interquartile range, *Lu-PSMA *Lutetium prostate-specific membrane antigen radioligand therapy, *mCRPC *Metastatic castration-resistant prostate cancer, *MDT *Metastasis-directed therapy, *mHSPC *Metastatic hormone-sensitive prostate cancer, *NA *Not available, *PCa *Prostate cancer, *PSA *Prostate-specific antigen, *RP *Radical prostatectomy, *RT *Radiation therapy

Of all included patients, 23% (*n* = 24) apalutamide-treated mHSPC patients had ≥ 10 bone metastases. Patients with ≥ 10 bone metastases harbored higher PSA level at treatment initiation (254 vs. 29 ng/ml) and achieved lower PSA response under treatment (PSA nadir 0.64 vs. 0.03 ng/ml). Moreover, patients with ≥ 10 bone metastases received less local therapy to the primary prostate tumor (4.2% vs. 37%) or metastasis-directed therapy (0% vs. 22%, all *p* ≤ 0.01). Concomitant visceral metastasis occurred in 4.5% of patients with ≥ 10 bone metastases and in 5.1% of patients with < 10 bone metastases (*p* > 0.9).

### ttCRPC with apalutamide: ≥ 10 vs. < 10 bone metastases

**Fig. 1:**
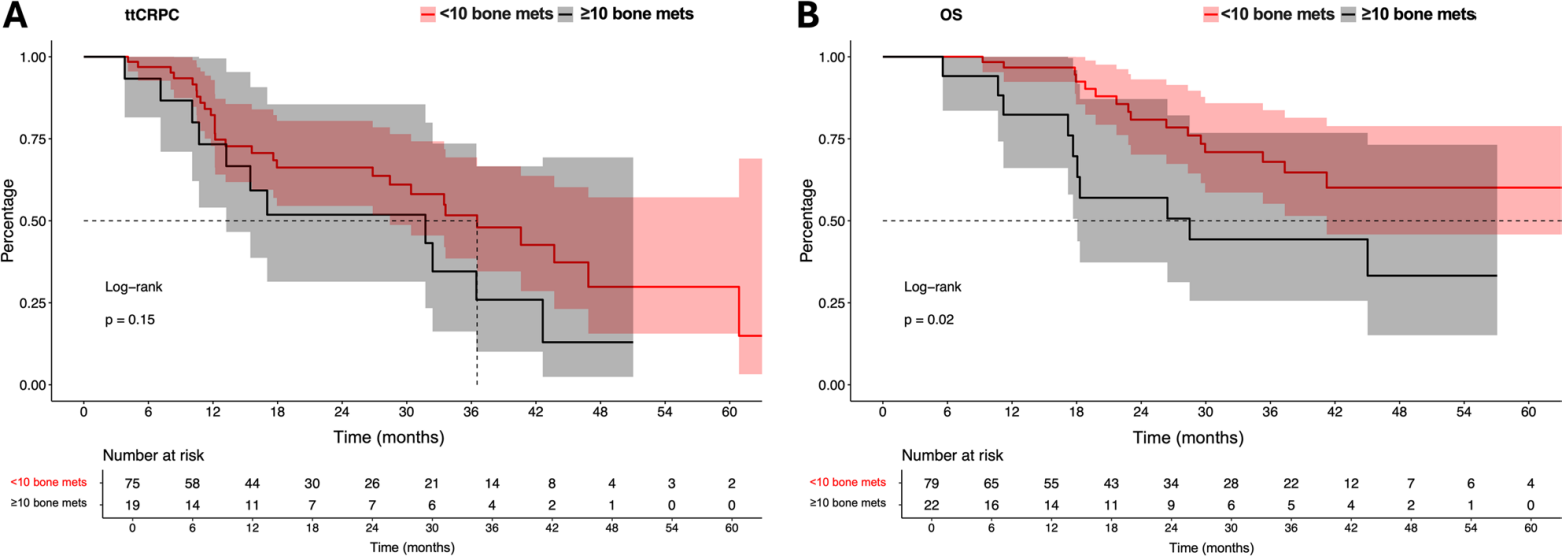
Kaplan Meier curves depicting time to metastatic castration resistant prostate cancer (ttCRPC) (**A**) and overall survival (OS) (**B**) in apalutamide metastatic hormone-sensitive prostate cancer (mHSPC) patients stratified according to ≥ 10 vs. < 10 bone metastases

Of the entire cohort, ttCRPC was 37 months. In ttCRPC analyses of 19 vs. 75 mHPSC patients with available ttCRPC information (Fig. 1A), no statistically significant difference in the comparison between the ≥ 10 vs. < 10 bone metastases groups were observed (*p* = 0.15), with median ttCRPC of 32 vs. 37 months for ≥ 10 vs. < 10 bone metastases. Corresponding HR was 1.67 (CI 0.83–3.37). 12- and 24-month mCRPC-free survival rates were 73.3% vs. 82.2% and 51.9% vs. 66.2% for ≥ 10 vs. < 10 bone metastases apalutamide-treated mHSPC patients. After further multivariable adjustment in Cox regression models, neither the presence of ≥ 10 bone metastases nor any of the control variables received independent predictor status of ttCRPC (Table 2).

**Table 2:** Univariable und multivariable Cox regression models predicting time to castration resistant prostate cancer (A) and overall survival (B)

	Univariable			Multivariable		
	**HR**	**CI**	***p*** **-value**	**HR**	**CI**	***p*** **-value**
**(A) ttCRPC**						
≥ 10 bone metastases	1.67	0.83–3.37	0.15	1.80	0.78–4.14	0.17
Age at mHSPC	1.02	0.99–1.05	0.13	1.00	0.95–1.07	0.9
Gleason score 8–10	1.35	0.75–2.43	0.3	0.99	0.38–2.61	> 0.9
De Novo mHSPC	1.63	0.92–2.88	0.10	1.90	0.24–15.26	0.6
ECOG status ≥ 1	2.19	1.11–4.31	0.02	1.69	0.58–4.94	0.3
**(B) Overall survival**						
≥ 10 bone metastases	2.49	1.13–5.49	0.02	3.34	1.11–10.07	0.03
Age at mHSPC	1.03	0.99–1.07	0.14	1.01	0.96–1.07	0.7
Gleason score 8–10	2.33	0.97–5.61	0.06	4.21	0.65–27.43	0.13
De Novo mHSPC	1.59	0.75–3.38	0.2	0.72	0.08–6.51	0.8
ECOG status ≥ 1	3.03	1.43–6.39	< 0.01	1.94	0.67–5.61	0.2
Systemic therapy lines for PCa	1.10	0.84–1.43	0.5	1.39	0.76–2.55	0.3

Additional variables were excluded due to collinearity and limited events per variable

*Abbreviations*: *CI *Confidence interval, *ECOG *Eastern Cooperative Oncology Group, *HR *Hazard Ratio, *mHSPC *Metastatic hormone-sensitive prostate cancer, *PCa *Prostate cancer, *ttCRPC *Time to metastatic castration-resistant prostate cancer

### OS with apalutamide: ≥ 10 vs. < 10 bone metastases

In the entire cohort, median OS was not reached, with 24- and 48-month OS rates of 80% and 62%, respectively. In OS analyses of 22 vs. 79 mHSPC patients with available OS information (Fig. 1B), significant differences in the comparison between the ≥ 10 vs. < 10 bone metastases groups were observed (*p* = 0.02), with median OS of 29 vs. 64 months for ≥ 10 vs. < 10 bone metastases. Corresponding HR was 2.49 (CI 1.13–5.49). Median 24- and 48-month OS rates were 59.5% vs. 83.3% and 47.6% vs. 61.8% for ≥ 10 vs. < 10 bone metastases apalutamide-treated mHSPC patients. After further multivariable adjustment in Cox regression models, the presence of ≥ 10 bone metastases was identified as an independent predictor for worse OS with a HR of 3.34 (CI: 1.11–10.07, *p* = 0.03; Table 2).

### ttCRPC with ≥ 10 bone metastases: apalutamide vs. abiraterone vs. docetaxel

**Fig. 2:**
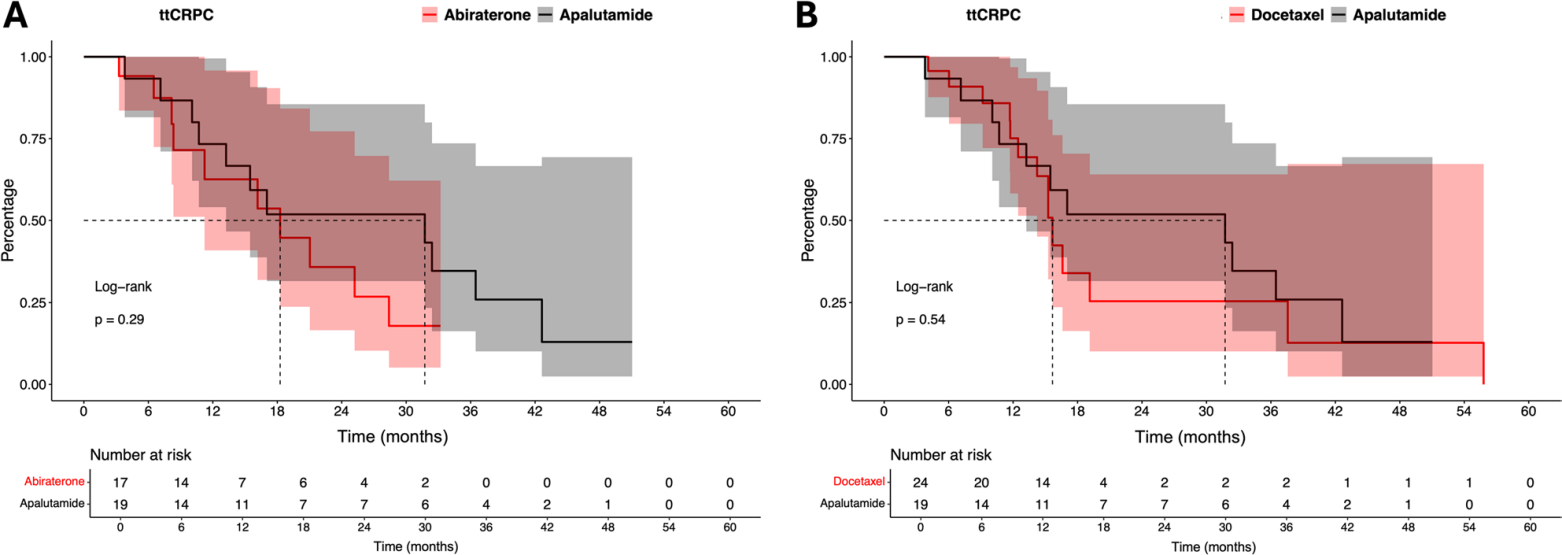
Kaplan Meier curves depicting time to metastatic castration resistant prostate cancer (ttCRPC) treated with apalutamide vs. abiraterone (**A**) and vs. docetaxel (**B**) in metastatic hormone-sensitive prostate cancer patients with ≥ 10 bone metastases

Further exploratory analyses for comparisons regarding ttCRPC in mHSPC patients with ≥ 10 bone metastases treated with apalutamide vs. abiraterone (*n* = 19 vs. *n* = 17) and apalutamide vs. docetaxel (*n* = 19 vs. *n* = 24) were made (Fig. 2, Table S1). Here, median ttCRPC was 32 vs. 18 months for apalutamide vs. abiraterone. Moreover, in comparison of apalutamide vs. docetaxel, median ttCRPC was 32 vs. 16 months (both *p* > 0.05). Due to the limited sample size, these analyses should be interpreted as hypothesis-generating only.

## Discussion

In this real-world analysis, we evaluated whether cancer-control outcomes in apalutamide-treated mHSPC patients differ according to bone metastatic burden. In addition, we explored potential differences in outcomes between patients with extensive bone metastases treated with apalutamide and those receiving other approved mHSPC doublet therapies. Using the FRAMCAP database, we made several important observations.

First, when baseline disease characteristics of our real-world cohort were compared to the apalutamide-treated patients of the TITAN trial, important differences were observed [10]. While no mHSPC patients with ECOG performance status ≥ 2 were included into the TITAN trial, 8.0% of our overall study cohort and 14% of patients with ≥ 10 bone metastases harbored ECOG performance status ≥ 2. In addition, the initial PSA level of 46 ng/ml in the current overall cohort was significantly higher than 5.97 ng/ml in the apalutamide arm of the TITAN trial [10]. All of these observations suggests that more fragile as well as more advanced prostate cancer patients were treated within our current real-world study compared to the apalutamide arm of the TITAN trial. Thus, the overall prognosis is expected to be worse. This assumption is also reflected in the median ttCRPC in our cohort, which was 37 months whereas in the apalutamide arm of the TITAN trial it had not yet been reached at the 44-month follow-up. However, the OS rates at 48 months of follow up, were 62% in the current overall cohort vs. 65% in the apalutamide arm of the TITAN trial, and therefore quite comparable [26]. Other key characteristics of the current study cohorts were very similar, including age, Gleason score proportions or rates of local therapy to the prostate, as well as the PSA nadir under apalutamide treatment. The mCRPC-free survival and OS rates in our overall cohort were comparable to those from other published real-world cohorts of apalutamide-treated mHSPC patients [27–29].

Moreover, 24% percent of included apalutamide-treated patients within the current real-world study harbored ≥ 10 bone metastases at mHSPC diagnoses, which is substantially lower compared to 38.3% in the post-hoc analysis cohort of the TITAN trial and provides important information regarding differences in patient selection for phase III trials and real-world cohorts [22].

Second, when cancer-control outcomes of apalutamide-treated mHSPC patients stratified according to number of bone metastases were compared, important findings were made. Specifically, no statistically significant difference regarding ttCRPC in the comparison between ≥ 10 vs. < 10 bone metastases apalutamide-treated mHSPC patients was observed although patients with ≥ 10 bone metastases had higher baseline PSA levels and lower PSA responses under treatment. However, after additional adjustment for these baseline patient and tumor characteristics, also no difference was observed, providing statistical robustness and clinical meaningfulness of the current findings. In OS comparisons, significant differences between ≥ 10 vs. < 10 bone metastases apalutamide-treated mHSPC patients were observed, even after multivariable adjustment for baseline patient and tumor characteristics. Therefore, our data confirm that the burden of bone metastases significantly impacts clinical outcomes especially for OS, the main and most important endpoint for clinicians and patients in treatment selection. These findings underscore the clinical relevance of stratifying mHSPC patients according to the count of bone metastases [1–5]. As a proxy for ttCRPC, post-hoc radiologic progression-free survival analyses from the TITAN trial reported a median of 23.4 months in apalutamide-treated mHSPC patients with ≥ 10 bone metastases, which is shorter than observed in our cohort, while median OS was not reached [22]. Comparison with other previous randomized controlled trials with high metastatic burden are difficult, since most studies only rely on CHAARTED criteria for low vs. high volume mHSPC patients, in which patients need to harbor at least four bone metastases to be stratified as high volume [18]. Within our cohort of patients with ≥ 10 bone metastases, 100% fulfilled criteria for high volume mHSPC, also with an unfavorable proportion of approximately 5% of patients presenting with concomitant visceral metastases. Therefore, comparisons with high volume mHSPC stratified patients may underestimate the current findings since the average metastatic burden may be significantly higher compared to average high volume mHSPC patients. Nonetheless, our findings reveal that favorable cancer-control outcomes can be achieved with apalutamide even with extensively high bone metastatic burden. Differences in OS outcomes may be partially explained by differences in subsequent treatments which is supported by virtually similar ttCRPC findings in the current cohort of ≥ 10 vs. < 10 bone metastatic mHSPC apalutamide-treated patients.

Finally, when exploratory analyses were made against other currently available treatment options for mHSPC patients, also important findings were made. Especially, apalutamide-treated mHSPC patients with ≥ 10 bone metastases exhibited numerically longer ttCRPC (32 months), relative to abiraterone (18 months) and docetaxel patients (16 months) with ≥ 10 bone metastases. The ttCRPC achieved in our real-world cohort in patients with ≥ 10 bone metastases treated with abiraterone or docetaxel were comparable to those from other published real-world cohorts investigating patients with high bone metastatic burden or high-volume disease according to CHAARTED criteria ranging from 12.5 to 23 months [18, 30–32]. However, due to sample size limitations, these analyses can only be interpreted as exploratory and viewed as hypothesis-generating. Our results suggest that apalutamide is a viable treatment choice also for patients with extensive metastatic disease, despite the associated poor overall prognosis [1–5].

Limitations of the current study should be acknowledged in its interpretation, such as the retrospective single-center design. Our cohort may be subject to selection bias, as it represents a tertiary care center population and apalutamide may have been more frequently used in patients considered less suitable for chemotherapy +/– ARPI. Inherent to the real-world setting, some variables required for cohort stratification or selected analyses were incompletely documented in a subset of patients, resulting in varying sample sizes across analyses and the exclusion of some patients, specifically due to unknown numbers of bone metastases or dates of progression. No imputation strategies for missing data were applied to avoid introducing additional assumptions. Consequently, despite multivariable adjustment, residual confounding cannot be excluded. In particular, OS results must be interpreted with caution, as information on subsequent mCRPC treatments was not available for all patients. However, most patients received taxane-based chemotherapy or an ARPI switch, which may result in similar subsequent cancer-control outcomes [14]. Furthermore, limited sample sizes in selected subgroups may have reduced statistical power and affected multivariable analyses. Finally, sample sizes of patients receiving triplet therapy were limited in the FRAMCAP precluding a comprehensive comparison of triplet therapy regimens, which have emerged as a potential treatment option for high-volume mHSPC patients [6, 11–13, 18–21].

## Conclusions

This real-world study demonstrates that apalutamide provides effective cancer-control outcomes in mHSPC patients with high metastatic bone burden. Especially, ttCRPC remains comparable to those with lower metastatic burden, indicating that apalutamide effectively delays disease progression.

## Supplementary Information

Supplementary Material 1.

## Data Availability

The datasets used and/or analysed during the current study are available from the corresponding author on reasonable request.

## Abbreviations

mHSPC: Metastatic hormone–sensitive prostate cancer
PSA: Prostate–specific antigen
FRAMCAP: FRAnkfurt Metastatic CAncer database of the Prostate
ttCRPC: Time to metastatic castration–resistant prostate cancer
OS: Overall survival
mCRPC: Metastatic castration–resistant prostate
EAU: European Association of Urology
RECIST: Response Evaluation Criteria in Solid Tumors
IQR: Interquartile range
ECOG: Eastern Cooperative Oncology Group Performance Status
ARPI: Androgen receptor pathway inhibitor
ADT: Androgen deprivation therapy
HR: Hazard ratio
CI: Confidence interval
CT: Computed tomography
PSMA: PET–prostate–specific membrane antigen positron emission tomography
