# Supplementary material for: Cancer-control outcomes of patients with metastatic hormone-sensitive prostate cancer and ≥ 10 bone metastases receiving apalutamide: a real-world cohort
Source: BMC Cancer. 2026 Mar 2;26:452. doi: 10.1186/s12885-026-15803-y (PMC13063726; doi:10.1186/s12885-026-15803-y)
Supplement: Supplementary file 1 — Supplementary Material 1. [file 12885_2026_15803_MOESM1_ESM.docx]

**Supplementary Table S1.** Descriptive characteristics of metastatic hormone-sensitive prostate cancer patients with ≥10 bone metastases treated with apalutamide, abiraterone or docetaxel.

| Characteristic | N | Abiraterone  N = 18 | Apalutamide  N = 24 | Docetaxel  N = 24 |
| --- | --- | --- | --- | --- |
| Age at metastatic PCa (yr) | 66 | 69 (64, 75) | 72 (67, 79) | 65 (58, 74) |
| PSA at mHSPC (ng/ml) | 52 | 328 (111, 732) | 254 (84, 531) | 341 (36, 505) |
| PSA nadir at mHSPC (ng/ml) | 38 | 1.26 (0.61, 9.97) | 0.64 (0.08, 3.91) | 1.64 (0.35, 9.80) |
| PSA response ≥90% | 38 | 8 (100%) | 16 (100%) | 14 (100%) |
| PSA at mCRPC (ng/ml) | 24 | 86 (49, 318) | 16 (7, 20) | 96 (22, 131) |
| Systemic therapy lines for PCa | 66 | 2 (2, 2) | 2 (2, 2) | 2 (2, 4) |
| ECOG status | 60 |  |  |  |
| 0 |  | 8 (47%) | 14 (67%) | 9 (41%) |
| 1 |  | 7 (41%) | 4 (19%) | 13 (59%) |
| ≥2 |  | 2 (12%) | 3 (14%) | 0 (0%) |
| Cardiovascular disease | 52 | 3 (27%) | 9 (38%) | 4 (24%) |
| Gleason score 8-10 | 63 | 16 (89%) | 17 (77%) | 22 (96%) |
| De Novo mHSPC | 66 | 17 (94%) | 24 (100%) | 24 (100%) |
| Local therapy with RP/RT | 66 | 1 (5.6%) | 1 (4.2%) | 2 (8.3%) |
| MDT | 66 | 2 (14%) | 0 (0%) | 0 (0%) |
| Concomitant visceral metastasis at mHSPC | 65 | 2 (11%) | 1 (4.5%) | 4 (19%) |
| High-volume mHSPC | 62 | 17 (94%) | 21 (100%) | 21 (95%) |
| High-risk mHSPC | 62 | 18 (100%) | 22 (100%) | 22 (100%) |
| Treatment of mCRPC | 66 |  |  |  |
| Chemotherapy |  | 4 (22%) | 2 (8.3%) | 2 (8.3%) |
| ARPI |  | 2 (11%) | 5 (21%) | 10 (42%) |
| Lu-PSMA |  | 2 (11%) | 0 (0%) | 0 (0%) |
| Radium |  | 1 (5.6%) | 0 (0%) | 0 (0%) |
| None/Other/NA |  | 9 (50%) | 17 (71%) | 12 (50%) |

Data are presented as median (IQR) or n (%).

Abbreviations: ARPI = androgen receptor pathway inhibitors; ECOG = Eastern Cooperative Oncology Group; IQR = interquartile range; Lu-PSMA = lutetium prostate-specific membrane antigen radioligand therapy; mCRPC = metastatic castration-resistant prostate cancer; MDT = metastasis-directed therapy; mHSPC = metastatic hormone-sensitive prostate cancer; NA = not available; PCa = prostate cancer; PSA = prostate-specific antigen; RP = radical prostatectomy; RT = radiation therapy.
